# Wide field retinal imaging and the detection of drug associated retinal toxicity

**DOI:** 10.1186/s40942-019-0172-0

**Published:** 2019-12-12

**Authors:** Giulia Corradetti, Sara Violanti, Adrian Au, David Sarraf

**Affiliations:** 1 Retina Disorders and Ophthalmic Genetics, Stein Eye Institute, University of California, Los Angeles, 100 Stein Plaza, Los Angeles, CA 90095 USA; 2 Greater Los Angeles VA Healthcare Center, Los Angeles, CA USA

**Keywords:** Drug toxicity, Periphery, Ultra-widefield

## Abstract

**Background:**

To describe the peripheral retinal findings associated with systemic medication toxicity and to outline the importance of ultra-widefield imaging in the detection, analysis and monitoring of these abnormalities.

**Main text:**

This review highlights the retinal manifestations associated with the more common drug toxicities, with emphasis on the peripheral features and the indications for wide field imaging. The presenting findings, underlying pathophysiology, and retinal alterations in hydroxychloroquine, thioridazine, didanosine, tamoxifen, MEK-inhibitor, and immune checkpoint inhibitor associated drug toxicity will be described and the importance of wide field imaging in the evaluation of these abnormalities will be emphasized.

**Conclusions:**

Wide field retinal imaging can improve the detection of peripheral retinal abnormalities associated with drug toxicity and may be an important tool in the diagnosis and management of these disorders.

## Background

Modern medicine has revolutionized the management of systemic disorders with the introduction of drugs that may alter the natural disease course. However, the administration of drugs that are physiologically foreign to the body can lead to adverse side effects or toxicity with significant consequences. The retina is especially susceptible to the effects of systemic drugs. It has an extensive dual blood supply from the retinal and choroidal vasculature and is one of the most metabolically active tissues in the body with minimal ability to regenerate and is therefore at high risk of drug toxicity. Thus, it is of vital importance to patient safety that ophthalmologists evaluate and effectively monitor for adverse drug effects, especially those affecting the retina.

There has been a very rapid progression in the development of advanced retinal imaging systems that have dramatically improved the power of the ophthalmologist to detect and diagnose and better understand a wide spectrum of retinal disorders including those associated with systemic drug toxicity. Vigilance is necessary as adverse reactions can occur at any time during treatment or after drug discontinuation. Strategies to reduce the risk of toxicity have been developed with the introduction of powerful advanced retinal imaging tools that have led to the earlier detection of toxicity, timely drug withdrawal, and prevention of vision loss. This review will focus on the importance of ultra-wide field (UWF) imaging in the diagnosis of drug associated retinal toxicity and identification of peripheral retinal abnormalities associated with this disorder.

## Hydroxychloroquine (Plaquenil)

Hydroxychloroquine (HCQ), originally prescribed for malaria, is a very common treatment for autoimmune diseases, including rheumatoid arthritis, systemic lupus erythematosus, and other inflammatory and dermatologic disorders [1]. The risk of retinal toxicity, greater with chloroquine exposure, has been recognized for many years [2, 3]. Central visual field analysis and spectral-domain optical coherence tomography (SD-OCT) are considered the most effective tools for the early diagnosis of HCQ maculopathy before significant photoreceptor damage occurs [4–7].

The mechanism of HCQ toxicity is poorly understood. Histopathological studies have illustrated that early cytoplasmic changes are noted in the ganglion cells and photoreceptors with later involvement of the RPE [8]. HCQ is melanotropic and preferentially deposits in high melanin expressing tissue, such as the RPE [9]. When bound to melanin, HCQ may cause a slow, chronic and delayed toxicity possibly due to alterations in the lysosomal pH leading to the accumulation of lipofuscin, a toxic element associated with the development of age-related photoreceptor degeneration [10]. Studies have proposed that light absorption or cone metabolism may be involved given the localization of disease within the macula [9–13].

**Fig. 1:**
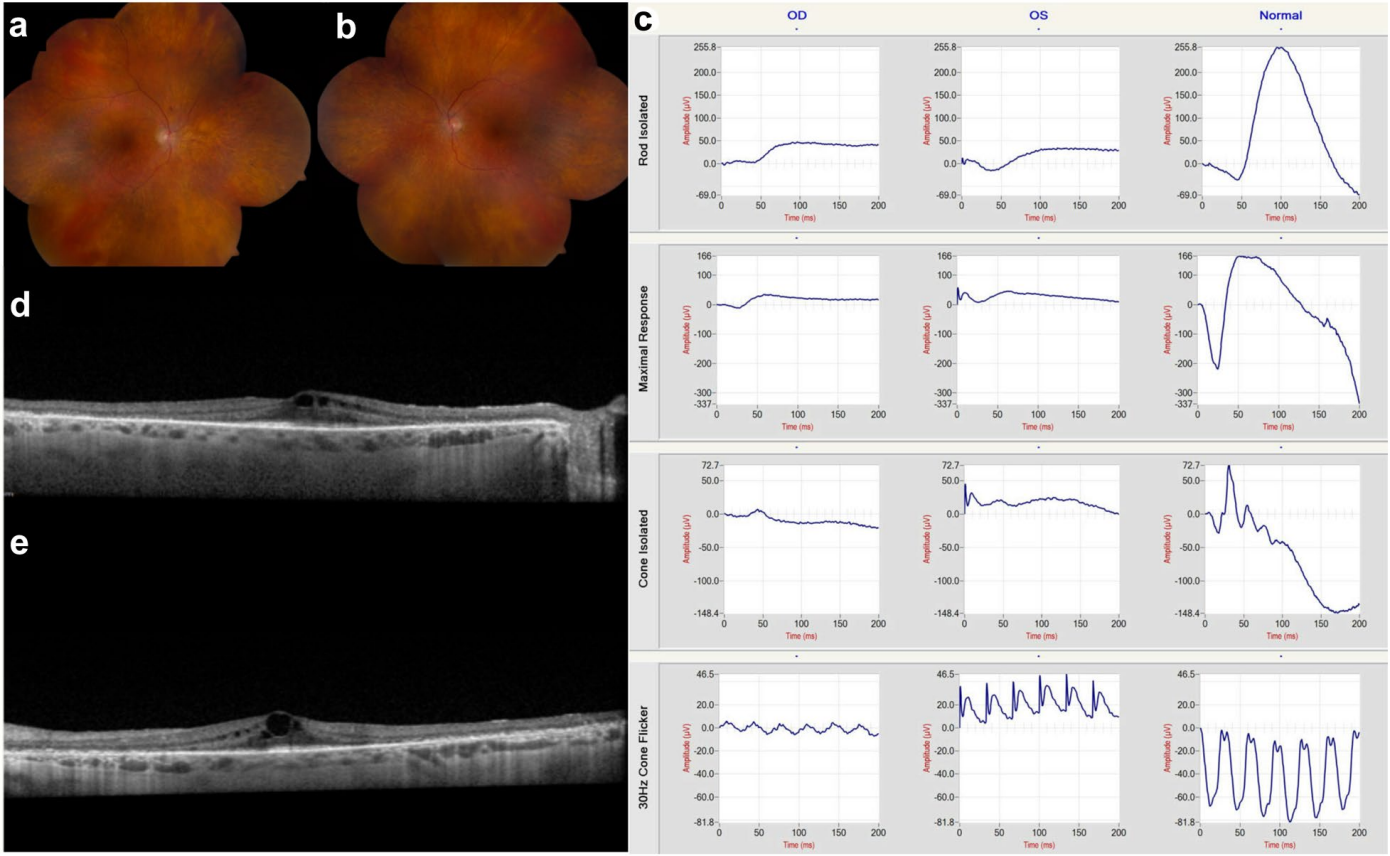
Hydroxychloroquine (Plaquenil). Diffuse retinal degeneration extending to the periphery associated with hydroxychloroquine retinal toxicity is illustrated with montaged color fundus photography (**a** and **b**). These findings were confirmed with full-field electroretinography which shows generalized depression of both rod and cone function in both eyes (**c**). The corresponding cross-sectional spectral domain-OCT illustrates the structural correlates of functional loss: there is diffuse pericentral ellipsoid zone loss associated with cystoid macular edema (**d** and **e**)

Toxic maculopathy is a potential side effect of long-term hydroxychloroquine therapy and the risk is dependent on a number of factors, including the cumulative dose, duration of use, weight-adjusted daily dose, associated tamoxifen therapy, and presence of concomitant kidney or liver disease [14–16]. Normally HCQ is excreted by the kidney or metabolized by the liver and persistent liver and renal dysfunction may potentiate its toxicity. Retinal toxicity in its earliest form starts as a focal area of parafoveal inner segment ellipsoid attenuation and then loss (especially inferotemporal) that may progress to develop the characteristic “flying saucer” sign with spectral domain OCT [7, 17]. With more advanced disease, a bull’s eye maculopathy may be identified with fundus autofluorescence or even color fundus photography associated with retinal pigment epithelium (RPE) disruption and atrophy [18]. If the medication is not discontinued, retinal toxicity may extend into the peripheral retina and a pan retinal degeneration may develop (Fig. 1) [13].

**Fig. 2:**
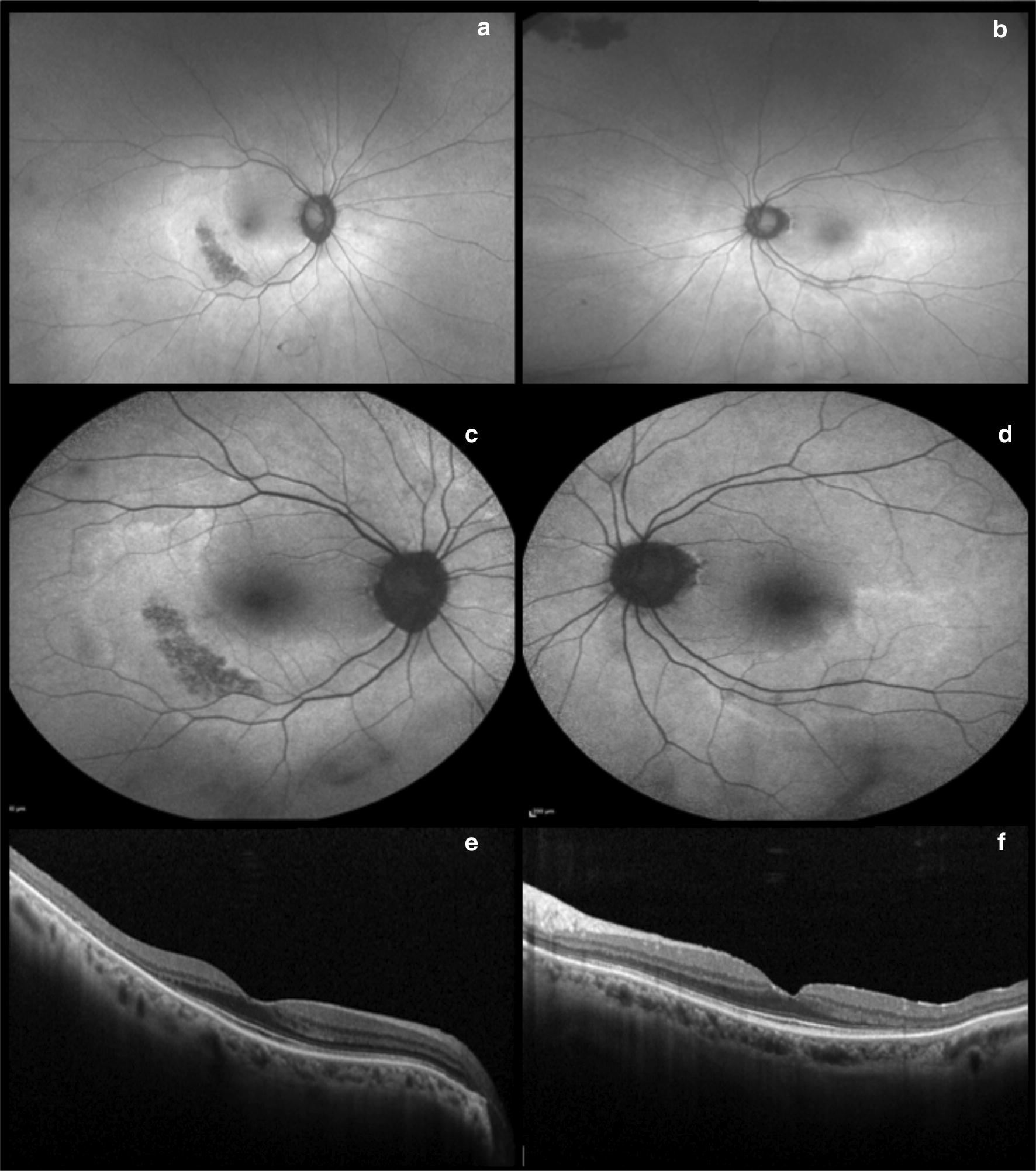
Hydroxychloroquine (Plaquenil). Optos ultra-widefield (**a** and **b**) and Heidelberg fundus autofluorescence (**c** and **d**) illustrate a more eccentric pericentral hyperautofluorescent ring corresponding to photoreceptor atrophy, sparing the fovea. Spectral domain-OCT displays bilateral and severe inner segment ellipsoid zone loss in the temporal perifovea (**e** and **f**)

**Fig. 3:**
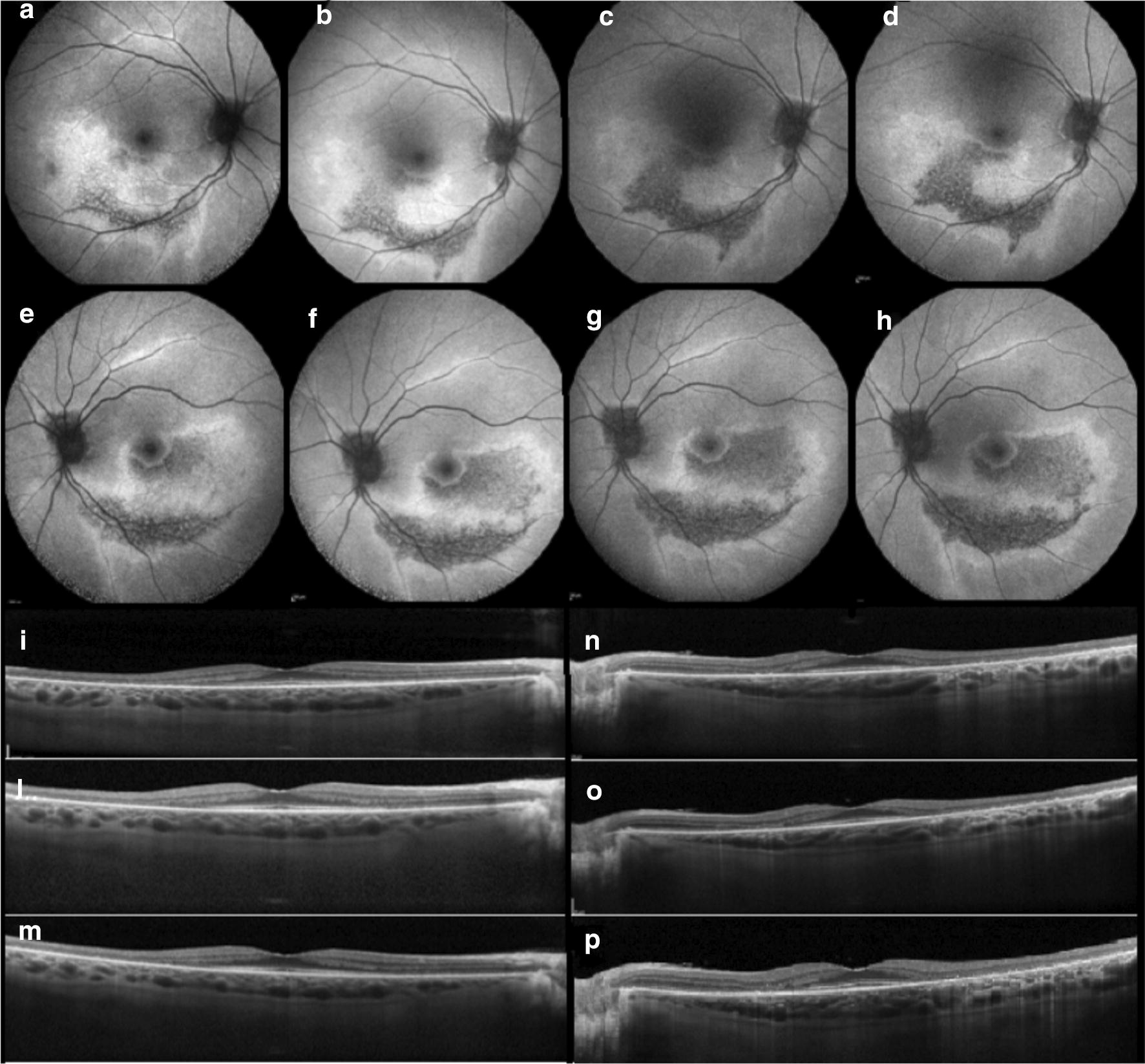
Hydroxychloroquine (Plaquenil). Wide angle fundus autofluorescence illustrates the progression of hydroxychloroquine retinal toxicity over time (despite discontinuation of the drug) in both eyes in the more eccentric pericentral distribution (**a**–**h**). Note the pathologic alterations displayed with spectral domain-OCT throughout the follow-up which include progressive outer retinal perifoveal thinning and loss of the ellipsoid zone band (**i**–**p**)

In Asian patients, a more peripheral toxicity may develop, even in the earlier stages of disease. The distinction between parafoveal and pericentral ellipsoid loss is important to recognize as Asian patients may manifest more peripheral retinal abnormalities facilitated with wide field imaging, specifically wide field fundus autofluorescence (Fig. 2). The more eccentric pericentral pattern of ellipsoid loss in Asian patients has been demonstrated in various studies [19, 20], including one that noted this pattern specifically in Korean patients. This was later confirmed in a large cohort using wide-field FAF in patients at high risk of HCQ toxicity and it was noted that 55% of Asian patients versus 2% of Caucasians displayed the more peripheral pericentral pattern and not the more classical parafoveal pattern. Therefore, wide field FAF may enhance the detection of HCQ toxicity especially in Asian patients (Fig. 3) [19, 20].

## Thioridazine (Mellaril)

Thioridazine (Mellaril) is an anti-psychotic phenothiazine derivative that was first clinically used in 1959. Shortly thereafter, the first case of ocular toxicity was described, a degenerative pigmentary retinopathy [21]. Toxicity with other phenothiazines such as chlorpromazine (Thorazine) is much rarer but the ocular findings including the retinal alterations are the same.

The underlying pathogenesis remains unclear but histopathologic studies have shown that phenothiazines like thioridazine bind melanin granules of the uvea and RPE for months. Primary toxicity of the uvea and RPE may lead to secondary loss of the choriocapillaris as RPE atrophy has been noted in areas of normal choriocapillaris perfusion [22]. Other studies have suggested that thioridazine exposure leads to inhibition of key retinal enzymes or binding to dopamine receptors, causing oxidative disruption of rhodopsin [21–25].

Toxicity has been typically reported at dosages greater than 800 mg/day over extended periods of exposure, with some studies reporting toxicity with dosages even less than 100 mg per day. Routine ocular examination with dilated retinal evaluation is necessary for patients receiving 600 mg per day of thioridazine or more [26–28]. Symptoms of more acute toxicity may develop due to very high dosages and include blurred vision, dyschromatopsia and nyctalopia, and typically occur 3–8 weeks after drug initiation. Visual acuity may be normal or variably reduced and color vision is often abnormal, especially with advanced cases. The visual field may display irregular paracentral or ring scotomas.

**Fig. 4:**
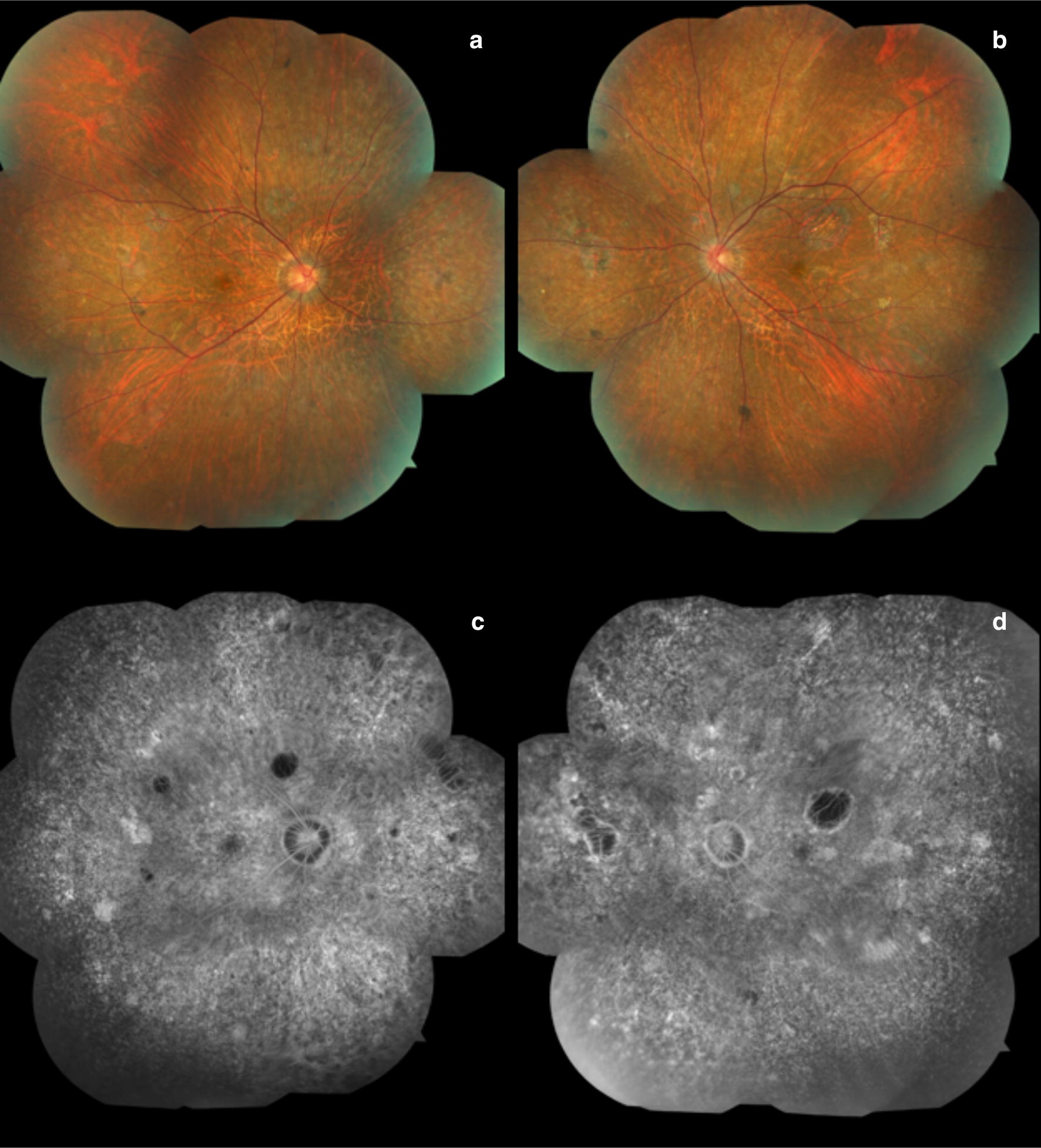
Thioridazine (Mellaril) Wide field color fundus photograph montage illustrates diffusely distributed pigment epithelial hyperplastic alterations and patches of retinal pigment epithelium atrophy throughout the fundus (**a** and **b**). Montaged wide field fluorescein angiography images display hypofluorescent patches and hyperfluorescent “window defects” associated with RPE atrophy with preservation of larger choroidal vessels (**c** and **d**)

Within weeks to a few months, a “salt and pepper” pigmentary retinopathy may acutely develop. Fine mottling or stippling of the RPE usually begins posterior to the equator but later clumps of coarse pigment may coalesce forming plaques (Fig. 4). Ultimately wide spread geographic or nummular RPE and choriocapillaris atrophy may ensue with chronic exposure. The ERG findings may include diffuse rod (and cone) dysfunction, which may return to normal after drug discontinuation [21, 29, 30].

Fluorescein angiography may illustrate widespread window defects of the RPE due to atrophy [25, 29, 31, 32]. Wide field fluorescein angiography and/or wide field FAF may be best to illustrate the disseminated pigmentary changes with acute toxicity or the diffuse nummular atrophic defects of the RPE and choriocapillaris with chronic exposure (Fig. 4). Pigmentary abnormalities of the retina may be irreversible and may illustrate progression after cessation of therapy, therefore early recognition of this pattern of retinal toxicity is very important [27, 31]. Significant visual acuity improvements have been reported after the medication is discontinued [33, 34].

Clinicians should be aware of the potential toxic retinal effects of thioridazine and should evaluate the retinal status and function before and during the treatment of psychiatric patients, especially those with vision changes. Multimodal imaging with UWF color photography, FAF and FA, in addition to functional tests such as visual field analysis and ERG testing, may be essential for early detection of the characteristic diffuse retinopathy which may guide selection of safer drug options and prevention of more widespread retinal damage and vision loss.

## Didanosine

The epidemic of acquired immunodeficiency syndrome (AIDS), caused by the human immunodeficiency virus (HIV), was first recognized in 1981 [35, 36]. Expedited clinical trials with antiretroviral drugs began in 1987 and zidovudine and didanosine (29,39-dideoxyinosine) were 2 of the first to show promise for the treatment of AIDS [37]. Both drugs are nucleoside reverse transcriptase inhibitors (NRTIs): didanosine is a purine adenine nucleoside analog while zidovudine is a pyrimidine thymine analog. Didanosine was approved by the FDA in 1991 for the combined treatment of HIV and was included in the breakthrough highly active anti-retroviral therapy (HAART) offered in 1996 [38, 39]. HAART therapy dramatically changed the prognosis and natural history of AIDS from certain untimely death to a chronic disease. This transition, however, harkened the development of drug toxicity as patients were exposed to prolonged didanosine therapy.

Didanosine (DDI) inhibits DNA polymerase and disrupts mitochondrial activity and is therefore associated with a litany of mitochondrial-associated side effects, including myopathy and optic neuropathy, and is no longer a first-line drug used for treatment of HIV disease. Patients with didanosine-associated retinal toxicity may present with a history of chronic DDI therapy dating back to the nineties [40]. The typical fundus findings include sharply demarcated RPE and chorioretinal atrophy extending circumferentially between the ora serrata and the posterior pole [41, 42]. DDI toxicity has funduscopic and FAF features very similar to the mitochondrial disorders (e.g. chronic progressive external ophthalmoplegia or CPEO, maternally inherited diabetes and deafness or MIDD and mitochondrial myopathy, encephalopathy, lactic acidosis and stroke or MELAS) that should be ruled out by careful history and genetic testing. The pattern of disease is nearly identical, with highly symmetric and concentric ring like areas of RPE mottling and/or chorioretinal atrophy, although the genetic syndromes occur in the macula while the drug-induced cases due to DDI occur in the periphery.

**Fig. 5:**
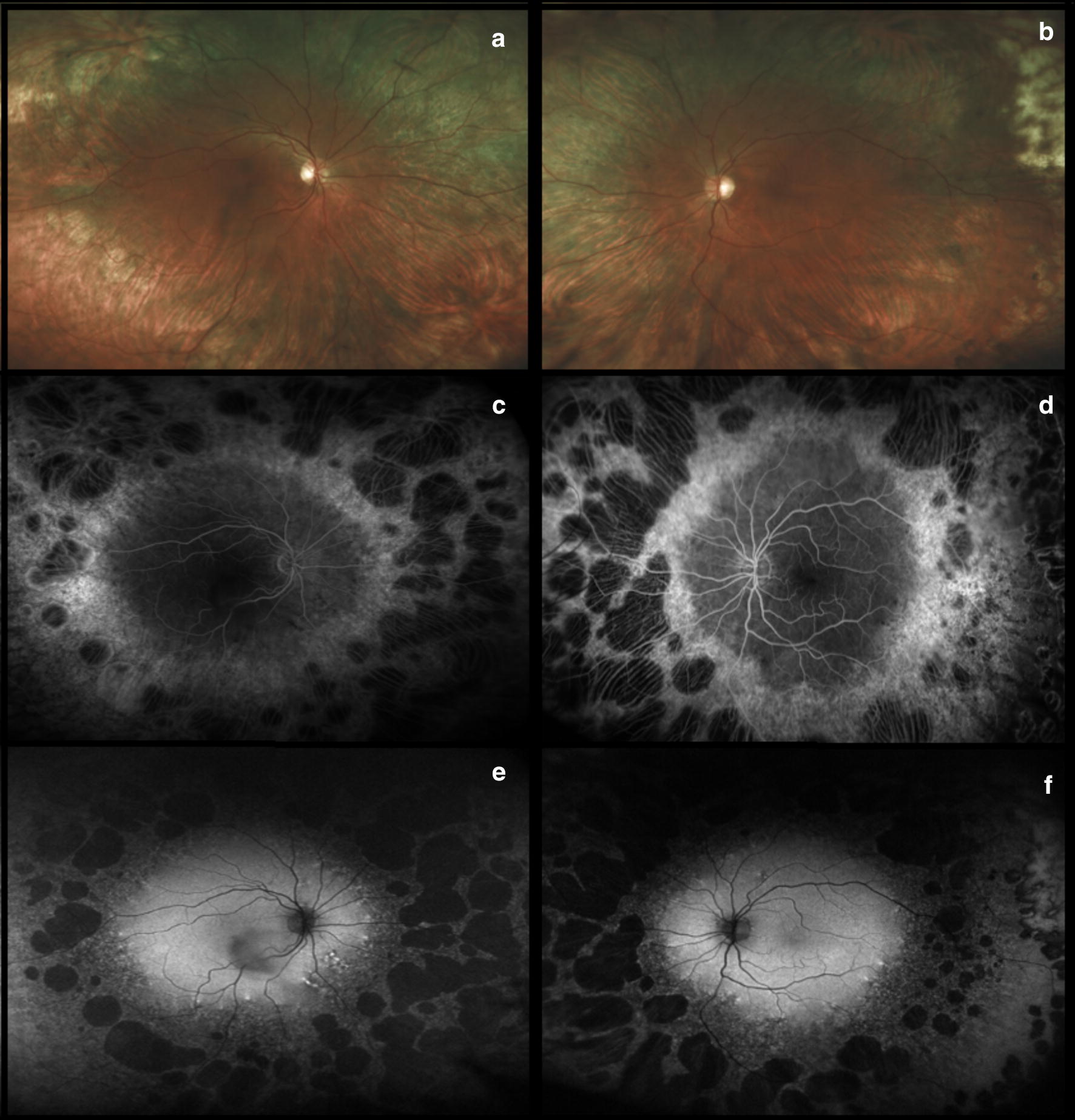
Didanosine. Ultra-widefield fundus photographs illustrate diffuse peripheral chorioretinal atrophy and sparing of the posterior pole (**a** and **b**). This atrophy is confirmed by the presence of large nummular bilateral areas of geographic atrophy on fluorescein angiography (**c** and **d**) and fundus autofluorescence (**e** and **f**). Images provided courtesy of Scott R Sneed M.D. and through permission from Haug SJ, Wong RW, Day S, Choudhry N, Sneed S, Prasad P, Read S, McDonald RH, Agarwal A, Davis J, Sarraf D. Didanosine retinal toxicity. Retina 2016;36 Suppl 1:S159-S167

In 1992 Whitcup et al. [41, 43] reported three children who developed bilateral and symmetric, well circumscribed peripheral chorioretinal atrophy after the initiation of didanosine therapy. Even with lower dosages of the drug, continued progression of the chorioretinal degeneration was noted in 2 of the cases. The study was expanded to include 95 children, with only one additional patient developing the characteristic peripheral chorioretinal degeneration [41, 43]. A very similar pattern of DDI associated peripheral chorioretinal degeneration was also reported in adult HIV patients many years later [44]. Haug et al. [45] published the largest series to date of nine cases of peripheral chorioretinal degeneration secondary to didanosine toxicity in adults. The fundus findings again consisted of concentric mid-peripheral and peripheral chorioretinal atrophy and degeneration, symmetrically present in each eye and sharply demarcated anterior to the posterior pole. A spectrum of alterations was noted ranging from diffuse retinal pigment epithelial (RPE) mottling to severe patches of geographic or nummular atrophy, and are best detected with wide field retinal imaging (Fig. 5) [45].

Histopathological analysis of an eye with didanosine toxicity performed after HIV-related death suggested that DDI primarily causes atrophy of the choriocapillaris and RPE with secondary loss of the overlying photoreceptors and outer retina. The mechanism of didanosine activity results from the NRTI inhibitory effects on mitochondrial DNA (mtDNA) and specifically, mtDNA polymerase. (46–48) DDI associated disruption of mtDNA polymerase may lead to cell- and tissue-specific deficiencies of mitochondrial driven oxidative phosphorylation, and may explain the progression of chorioretinal toxicity despite drug cessation [49, 50]. Further investigation is necessary to elucidate the remarkable peripheral phenotype of DDI associated retinal toxicity [44, 51].

Newer versions of HAART therapy contain tenofovir and emtricitabine, both NRTIs that are much safer alternatives to didanosine. However, tenofovir, may have a potentiating effect on mtDNA depletion and may compound the progressive toxic effects of didanosine on the peripheral chorioretinal complex [52]. Further research is needed to establish the relationship between these drugs and peripheral retinal degeneration. The mid-peripheral concentric, symmetric and sharply demarcated pigment epithelial mottling with chorioretinal atrophy can be considered a signature phenotype associated with exposure to DDI. This is a macular sparing disorder and therefore the presentation of didanosine retinal toxicity can be delayed or missed. An accurate retinal examination with multimodal imaging including wide-field FAF or wide field FA may be essential to detect the remarkable peripheral abnormalities and monitor patients treated with NRTI drugs.

## Tamoxifen

Tamoxifen citrate is currently the only FDA-approved selective estrogen receptor modulator for the adjuvant therapy of early-stage estrogen positive breast cancer. Tamoxifen is mainly a prodrug as two of its many metabolites, notably 4-hydroxy-N-desmethyltamoxifen (endoxifen) and also 4-hydroxytamoxifen (4-OHT), are known to have greater affinity for the estrogen receptor than tamoxifen itself. Routine therapeutic dosage starts at 20 mg daily and can be increased up to 40 mg daily. For chemotherapeutic indications, e.g. malignant astrocytoma of the brain, tamoxifen dosages can exceed 200 mg daily and the risk of retinal toxicity is therefore much greater. Ocular features are generally observed after a daily dose greater than 120 mg or a cumulative dose greater than 100 g [53, 54].

Gallicchio et al. [55] was the first to note possible tamoxifen toxicity in the eye. In the initial report, 13 of 97 tamoxifen users self-reported non-specific vision problems. When evaluating these 13 women, the authors noted significantly higher serum concentrations of tamoxifen and N-desmethyltamoxifen (N-DMT), a hydroxylated endoxifen, than the 84 women who did not report visual disturbances [55].

**Fig. 6:**
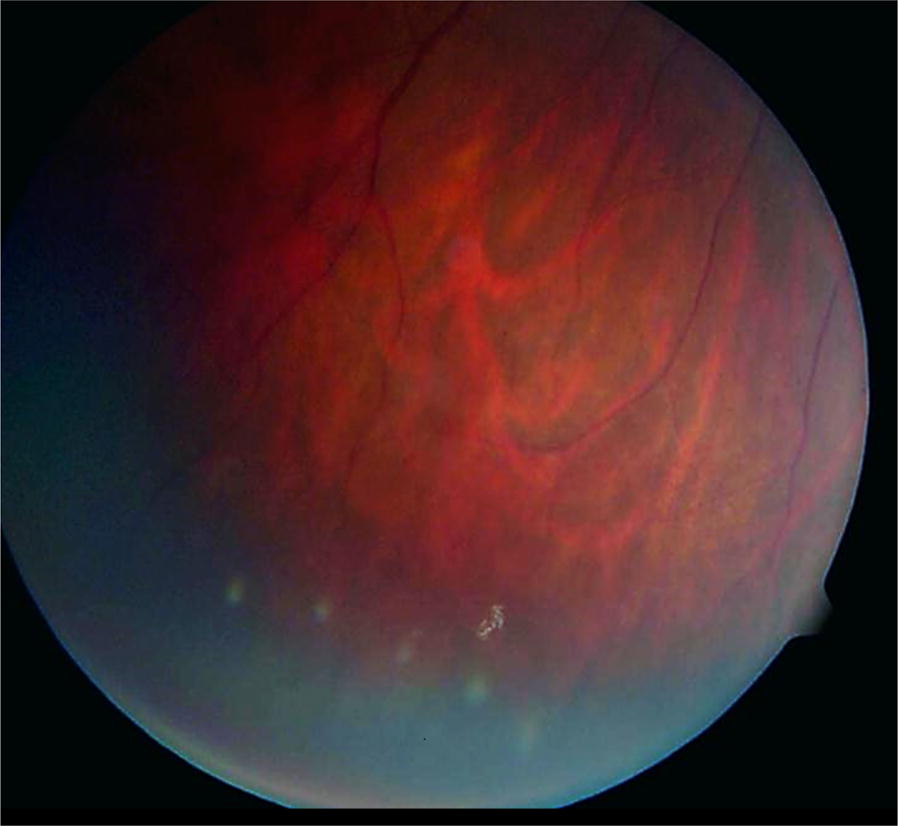
Tamoxifen. Peripheral color fundus photograph of the left eye illustrates multiple white refractile deposits in the inferotemporal periphery. Images provided through permission from Bourla DH, Sarraf D, Schwartz SD. Peripheral retinopathy and maculopathy in high-dose tamoxifen therapy. Am J Ophthalmol. 2007;144(1):126–8

Well documented tamoxifen induced toxicity includes keratopathy, cataract and optic neuritis but the most potentially devastating side effect is the development of crystalline retinopathy associated with cystoid macular edema (CME) [56–59]. The brilliant intraretinal crystals typically cluster within the perifoveal macular region with a characteristic annular distribution that may vary in density. The crystals appear to be confined to the nerve fiber layer and inner plexiform layer based on optical coherence tomography (OCT) imaging of the retina [60]. However, vision decline is more commonly a consequence of CME, which can even occur in low cumulative doses [53, 54]. Crystals may improve or clear with discontinuation of systemic therapy and CME can resolve with anti-VEGF injections [56]. Crystalline toxicity is very rare with the more popularized safer dosages used for adjuvant breast cancer therapy. At these lower dosages however, ellipsoid loss or inner or outer retinal cavitation in the fovea, similar to the findings of Macular Telangiectasia Type 2, have been very rarely reported (Fig. 6).

Tamoxifen can cause retinal damage through multiple mechanisms, but the two most studied are related to direct toxicity to retinal cells and their off-target effects on Müller glia. First, the cationic and amphophilic properties of tamoxifen can cause drug-polar lipid complexes to accumulate in lysosomes, inducing cellular oxidative damage [61]. Second, tamoxifen inhibits the glutamate-aspartate transporter in Müller cells that are vital in maintaining retinal cell integrity and homeostasis [62]. This causes excessive intracellular glutamate build-up that results in Müller cell dysfunction and apoptosis, vascular remodelling, and neurodegeneration of the retinal layers [62]. These mechanisms explain the propensity for central macular disease, where both blood flow (and therefore drug levels) and Müller cell density are the greatest [63, 64].

Peripheral retinal toxicity has rarely been reported in association with tamoxifen exposure. Bourla et al. [65] noted the presence of peripheral retinal crystals with wide field color fundus photography in patients with tamoxifen maculopathy after high dose chemotherapy for brain tumors [65]. These findings may be more common than previously reported in patients with high-dose tamoxifen therapy and may be missed without the benefit of advanced wide field imaging technologies (Fig. 6).

## BRAF/MEK inhibitors

Immunomodulation and targeted blockades of regulatory growth signaling pathways have been recently introduced for the management of advanced cutaneous melanoma management. Fundamental understanding of the molecular mechanisms and oncogenesis of melanoma has led to the development of inhibitors that have significantly extended the survival of patients with metastatic cutaneous melanoma [66–69]. Briefly, mutations in BRAF, an up-stream regulator of mitogen-activated protein kinase enzymes (MEK), causes dysregulated activation of MEK, promoting cellular proliferation and cancer through the well-described RAS-MEK-ERK1/2 pathway. FDA approved BRAF inhibitors for metastatic melanoma include Vermurafenib, Dabrafenib, Trametinib, which target BRAF V600E or V600K, while Cobimetinib specifically inhibits MEK [66–69]. Currently, similar molecules and additional studies are being conducted to evaluate the efficacy in various other cancers [70]. Therefore, the incidence of BRAF/MEK inhibitor induced toxicity may rise in the future.

BRAF/MEK inhibitors constitute a burgeoning field in oncology but preliminary data suggest that these medications are associated with high rates of ocular toxicity. Typical systemic adverse events related to these drugs include skin-related toxicities and gastrointestinal disorders [71]. Common ophthalmologic side effects include chorioretinopathy and exudative retinal detachment [72]. Multiple case reports or series have reported the development of sub-foveal neurosensory retinal detachment occurring within a few hours to 4 weeks of starting treatment [44, 45, 49, 73]. Various patterns of subretinal fluid have been described resembling central serous chorioretinopathy (CSCR). Francis et al. [74] have provided clinical guidelines using multimodal retinal imaging to differentiate MEK inhibitor retinal toxicity from CSCR. Of note, in a separate study a dose-dependent increase in retinal volume and central retinal thickness were observed during the first weeks of treatment. These alterations resolved gradually over three to 6 months without any apparent functional deficits or change in structural retinal integrity [75].

The pathophysiological mechanism BRAF/MEK inhibitor induced retinal toxicity has been proposed to involve dysfunction of the retinal pigment epithelium (RPE), which has important metabolic and fluid regulation activities [42, 43]. Jiang et al. [76] demonstrated that aquaporins, membrane proteins that control fluid transport from the RPE cells, are mediated by the MEK/ERK pathway, and inhibition therefore disrupts fluid dynamics at the outer blood ocular barrier level. In addition, Schoenberger et al. [77] reported that MEK inhibition may induce oxidation and inflammation altering the permeability of the RPE.

**Fig. 7:**
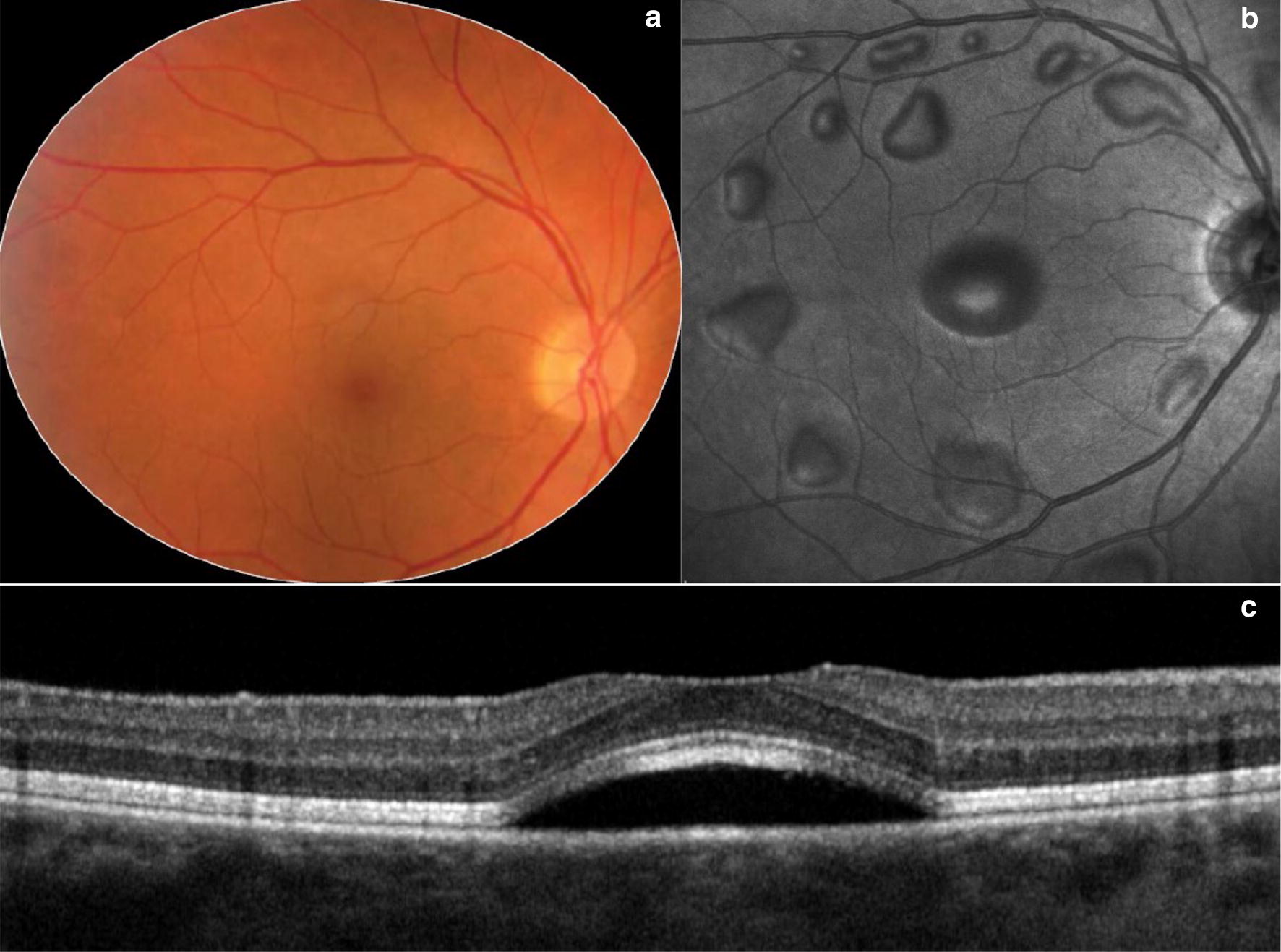
MEK inhibitor. Wide angle color fundus photograph (**a**) and en-face near infra-red image (**b**) of the right eye from a patient treated with MEK inhibitor illustrates multiple serous retinal detachments. Spectral domain-OCT (**c**) through the fovea in an unrelated patient displays the characteristic sub-foveal serous retinal detachment due to MEK inhibitor toxicity. Images provided courtesy of Giuseppe Querques M.D. and Enrico Borrelli M.D.

BRAF/MEK inhibition retinal toxicity has a broad clinical phenotype which may require UWF imaging. MEK inhibitor-associated retinopathy (MEKAR) has been documented to be bilateral, multi-focal, with fluid typically localizing between the RPE and the interdigitation zone, most frequently in the central macular region without concurrent RPE detachment (Fig. 7) [74]. However, within the largest cohort of patients with MEKAR, fourteen of the twenty-five patients presented with lesions outside the posterior pole, most commonly around the arcades [74]. These findings were recapitulated in a report of a woman treated with trametinib who was noted to have symmetrical vitelliform-like lesions along the vascular arcades associated with central neurosensory detachments [78]. An additional paper reported acute exudative paraneoplastic polymorphous vitelliform maculopathy documented on UWF FAF that resolved with discontinuation of dabrafenib and trametinib [77].

As MEK inhibitors continue to grow in popularity for the treatment of cancer, a potential increase in incidence of associated ocular toxicities may occur. Although limited peripheral findings have been reported, peripheral lesions may develop and UWF retinal imaging may play a role in the detection and monitoring of MEK inhibitor toxicity [74].

## Immune checkpoint inhibitors (CPIs)

Immune checkpoint inhibitors (CPIs) have emerged as a powerful tool in the management of malignancy [79]. These drugs work at the level of the T cell and block innate inhibitory processes involving cytotoxic T-lymphocyte antigen-4 (CTLA-4), programmed death protein 1 (PD-1), and programmed death ligand-1 (PD-L1). Inhibition of the interaction between these inhibitory receptors and their ligands leads to an autoimmune like state with T cell activation that targets and kills tumor cells [80].

The list of novel CPIs is increasing. In 2011, the United States FDA approved ipilimumab, an IgG1 human monoclonal antibody antagonist of CTLA-4, for the treatment of metastatic melanoma. Nivolumab and pembrolizumab, antibodies against PD-1, have been approved for metastatic melanoma and non–small-cell lung cancer (NSCLC). Atezolizumab, avelumab, and durvalumab, which target PD-L1, have been approved for various different cancers [46, 47, 81–84]. These new treatments have demonstrated improved and durable responses, but have immune-related side effects which require prompt recognition and management distinct from traditional cytotoxic chemotherapies.

Ocular adverse effects secondary to checkpoint inhibitors are rare, reported in approximately 1% of patients and are related to upregulation of the immune system. The most commonly reported side effects include dry eye (1–24%), inflammatory uveitis (1%), and myasthenia gravis with ocular involvement. They occur typically within weeks to months of starting therapy, are rarely isolated, and often occur in conjunction with systemic immune-mediated adverse effects. Most autoimmune-like complications can be effectively managed with topical, periocular, or systemic corticosteroids [48, 85].

**Fig. 8:**
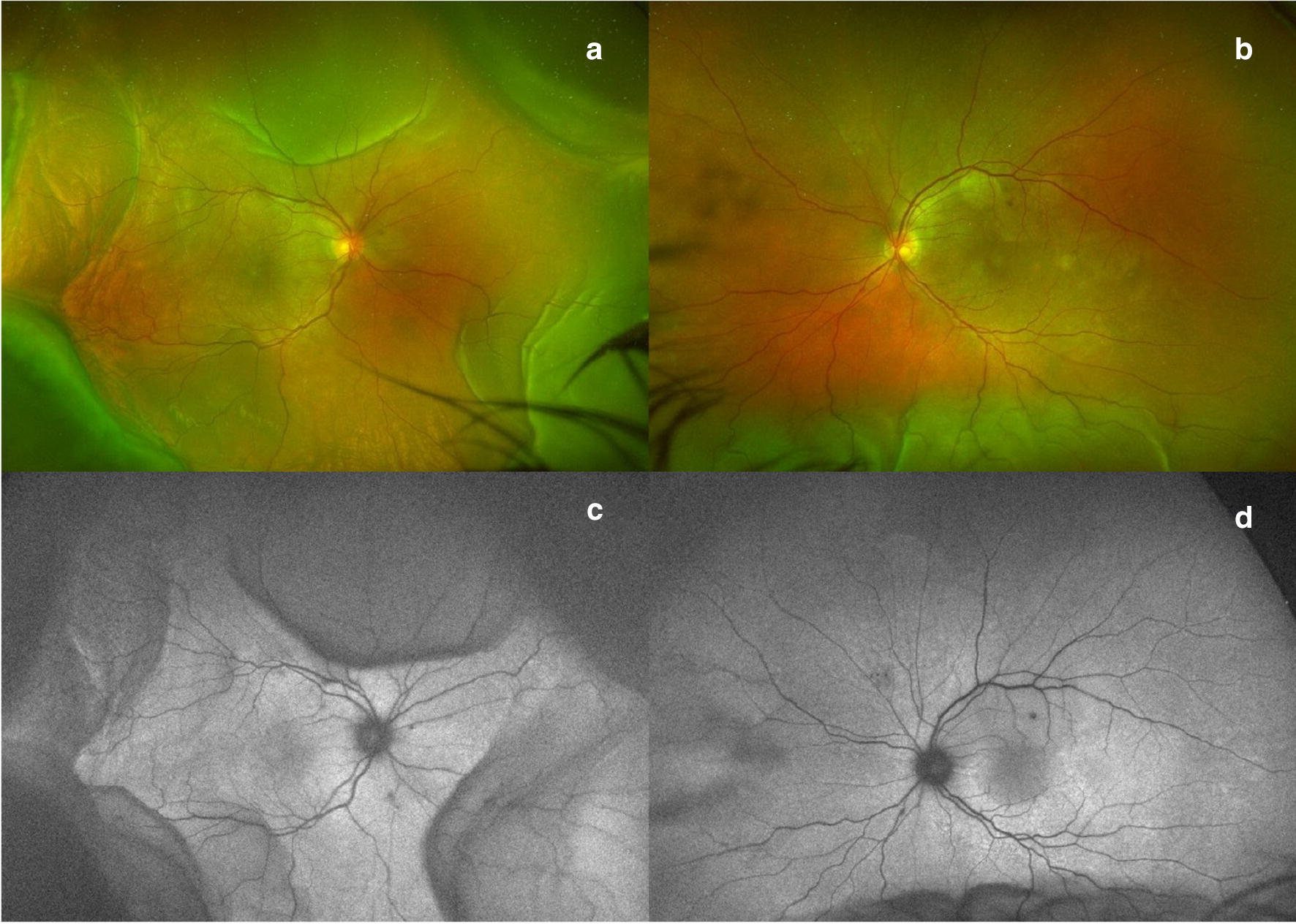
Checkpoint inhibitors. Ultra-widefield color fundus photograph (**a** and **b**) and fundus autofluorescence (**c** and **d**) from a patient with combination checkpoint inhibitor therapy (ipilimumab and nivolumab) illustrates ciliochoroidal effusions greater in the right than left eye. Images provided courtesy of Edmund Tsui M.D. and Yasha Modi M.D.

Retinal toxicities are beginning to be reported as these drugs gain more wide-spread use. UWF imaging in CPI toxicity can be an important resource to capture the remarkable peripheral retinal findings and to evaluate for peripheral leakage. Several case reports of patients with CPI retinal toxicity have described the presenting findings of uveitis or Vogt–Koyanagi–Harada (VKH)-like syndrome with peripheral leakage, responsive to corticosteroids [45, 86–89]. Intravitreal anti-VEGF therapy has shown some anecdotal success in eyes complicated by choroidal neovascularization [90]. One unique case report described bilateral severe choroidal effusion and exudative retinal detachment in a patient diagnosed with metastatic melanoma and treated with ipilimumab and nivolumab therapy, illustrating the importance of peripheral retinal imaging including wide field color photography and angiography (Fig. 8) [91]. Tsui et al. [91] hypothesized a synergistic effect of combined checkpoint inhibition.

These cases highlight the need for vigilance with multimodal imaging, specifically ultra- widefield fundus photography, fluorescein angiography and optical coherence tomography in identifying patients with CPI associated retinal toxicity. It is important to note that the findings presented are based largely on single case reports or small case series and additional studies are necessary to determine whether patient characteristics predispose to ocular side effects and which checkpoint inhibitors are at greatest risk for these side effects.

## Conclusions

Although the blood-retinal barrier prevents unlimited or indiscriminate exposure to systemic drugs, the high metabolic demands and inability to regenerate place the retina at high risk of toxicity. Drugs used for the management of systemic disease, such as those for autoimmune or infectious disease or malignancy, unfortunately have off-target effects that require monitoring. Having the knowledge and tools to identify, monitor, and intervene in cases with retinal drug toxicity is of vital importance. Diagnosis of retinal toxicity can be challenging especially when abnormalities are localized to the peripheral retinal quadrants and therefore wide field imaging, including wide field color photography, fundus autofluorescence and angiography, can be essential to facilitate early detection and early and appropriate drug discontinuation which can be vital to prevent irreversible or progressive retinal injury and vision loss.

As the incidence of ocular adverse events increases, especially in the era of rapid drug development, the use of multi-modal imaging, particularly ultra-widefield imaging for the identification of peripheral disease, will become more and more important in the diagnosis and management of drug toxicities of the retina.

## Data Availability

Data sharing is not applicable to this article as no datasets were generated or analysed during the current study.

## Abbreviations

UWF: ultra-widefield
SD-OCT: spectral domain optical coherence tomography
mfERG: multifocal electroretinography
FAF: fundus autofluorescence
FDA: food and drug administration
HCQ: hydroxychloroquine
N-DMT: N-desmethyl-tamoxifen
CME: cystoid macular edema
MEK: mitogen-activated protein kinase kinase enzymes
MAPK: ras-mitogen-activated protein kinase
HIC: human immunodeficiency virus
AIDS: acquired immunodeficiency syndrome
NRTIs: nucleoside reverse transcriptase inhibitors
mtDNA: mitochondrial DNA
CPIs: checkpoint inhibitors
CTLA-4: cytotoxic T-lymphocyte antigen-4
PD-1: programmed death protein 1
PD-L1: programmed death ligand-1
VKH: Vogt–Koyanagi Harada
